# Magnetic borylated conjugated polymer nanoparticles for deep-red optical and lifetime imaging

**DOI:** 10.1039/d6tb00192k

**Published:** 2026-04-01

**Authors:** Struan Bourke, Yurema Teijeiro Gonzalez, Patrick Bergstrom Mann, Daniel Luke Crossley, Laura Urbano, Lea Ann Dailey, Klaus Suhling, Michael L. Turner, Michael J. Ingleson, Mark Green

**Affiliations:** a Department of Physics, King's College London Strand Campus London WC2R 2LS UK mark.a.green@kcl.ac.uk; b School of Chemistry, University of Manchester Manchester M13 9PL UK; c Institute of Pharmaceutical Sciences, King's College London Waterloo Campus London SE1 9NH UK; d School of Health, Medicine and Life Sciences, School of Life and Medical Sciences, University of Hertfordshire Hatfield AL10 9AB UK; e Department of Pharmacy, University of Vienna, Josef-Holaubek Platz2 (UZA II) 1090 Vienna Austria; f Department of Chemistry, University of Edinburgh, Joseph Black Building David Brewster Road Edinburgh EH9 3FJ UK

## Abstract

The near-IR emitting conjugated polymer (borylated poly(9,9-dioctylfluorene-*alt*-benzothiadiazole)) was processed into colloidally stable nanoparticles doped with superparamagnetic iron oxide nanoparticles (SPIONs), giving multifunctional luminescent and magnetic nanoparticles between 100–120 nm in diameter. Emission between 710 and 720 nm was observed, and quantum yields (QYs) of up to 2.7% were achieved. The resulting particles were used to image HeLa cells using conventional and lifetime imaging techniques.

## Abstract 2

In this communication, we report the development of water-dispersible, deep-red emitting conjugated polymer based nanoparticles for multimodal optical and MR imaging.

## Graphical abstract

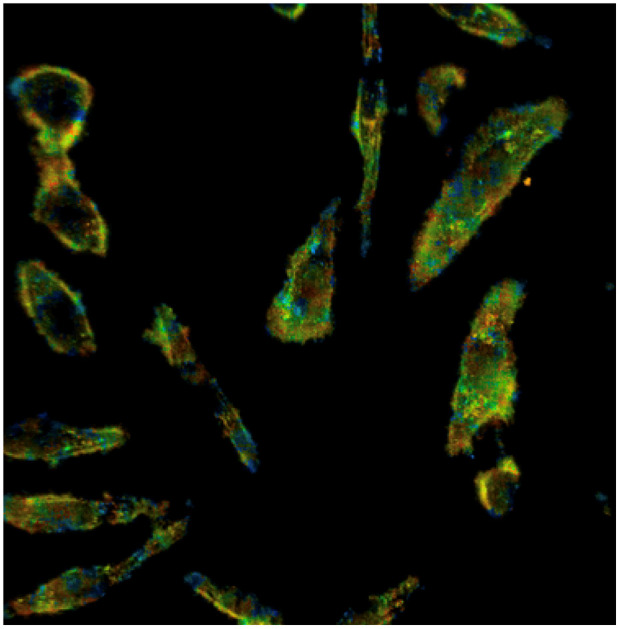

In the field of diagnostics, imaging agents that focus on higher image resolution, longer retention times, and the capacity for multimodal imaging can enhance patient care.^1^ However, for this to be fully realised, new imaging agents need to be developed and used with current imaging techniques. Conjugated polymers (CPs) are of interest as they can be used for a variety of medical applications, such as fluorescence and photoacoustic imaging^2,3^ as well as photothermal and photodynamic therapies.^4,5^ Compared to fluorescent dyes and quantum dots, π-conjugated polymers can possess larger absorption cross-sections, brighter emission and high photostability combined with a non-toxic composition.^6–11^

To make the CP materials suitable for biological applications, the polymers need to be dissolved or dispersed in aqueous media. This can be achieved using a range of functional amphiphilic polymers, ligands and biological molecules as capping or encapsulating agents. Abelha *et al.*^12^ and Kemal *et al.*^6^ have both reported that π-conjugated polymers can be embedded within self-assembling polymer micelles comprised of the copolymer of polylactic-*co*-glycolic-polyethylene glycol (PLGA–PEG) at extremely high production yields. Others have reported the use of cysteine-rich amphiphilic proteins as capping agents.^13^ Whilst these materials show the potential of CPNs (conjugated polymer nanoparticles), the majority emit in the visible region of the spectrum and there remains a desire for materials that emit in the biological spectral windows beyond this, *i.e.*, in the near-IR region. Previously, Crossley *et al.*^14^ developed a series of borylated CPs that emitted in the deep red region, which were then processed into small, photostable, bright silica-based nanoparticles. However, silica shells grown in this manner were difficult to modify to incorporate additional functional groups. It would be attractive to combine bright near-IR (with emission maximum >700 nm) emission with magnetic properties in a single water-dispersible nanoparticle.

The inherent hydrophobic nature of CPs makes them unusual as precursors for biologically relevant nanoparticles, but it allows other functional components to be added during nanoparticle preparation to create multimodal imaging agents, as reviewed by Farah *et al.*^15^ Whilst the majority of CPNs report only a single imaging modality, a small yet growing number of reports describe the engineering and preparation of multimodal particles, which can be utilised in a number of imaging applications. For example, in 2010, Howes *et al.*^16^ reported the preparation of nanoparticles comprised of π-conjugated polymers and superparamagnetic iron oxide nanoparticles. The optical properties from the resulting bimodal nanoparticles covered the entire visible range whilst maintaining bright emission and the inherent magnetic characteristics of the iron oxide. Notably, Xu *et al.* also reported the synthesis of a conjugated polymer with a Gd unit grafted as a side chain, although this was not processed into particles, nor the optical properties reported.^17^ This work did, however, highlight the versatility of conjugated polymers and their potential in imaging. Pan *et al.* used similar Gd chelates on the surface of near-IR emitting conjugated polymer particles to image mice bearing oral squamous cell carcinomas by both MRI and optical imaging.^18^ This additional modality is, however, relatively expensive whilst not having the advantages inherent in magnetic materials. Zhang *et al.* further developed the Fe_3_O_4_/polymer nanocomposites into smaller (between 35 nm and 75 nm) particles suitable for sentinel lymph node imaging, however the emission wavelength was at a maximum of *ca.* 550 nm, not suitable for detailed deep tissue imaging.^19^ Similar materials were used in the effective imaging of heterotopic brain tumours, using both optical and MRI techniques, however orthoptic models were not imaged as successfully.^20^

Here, we report the synthesis of poly(styrene-*co*-maleic anhydride), (PSMA) passivated deep red-emitting (emission maximum >700 nm) conjugated polymer nanoparticles, approximately 110 nm in diameter which incorporated superparamagnetic iron oxide nanoparticles (SPIONs) for potential multimodal imaging. The advantage of this work is the relative ease involved with modifying a commercially available conjugated polymer rather than synthesising a new polymer from first principle. This in effect takes an average yellow-emitting polymer, with little impact in biology, to a deep-red emitter that has potential applications in imaging. The role of amphiphilic polymers in the synthesis of conjugated polymer nanoparticles is twofold – to act as a passivating agent over the entire particle, creating a barrier between particles and inducing water solubility by the introduction of a polar group on the surface, and to provide a chemical functional group for targeting moiety attachment.^21^

## Results and discussion

**Fig. 1:**
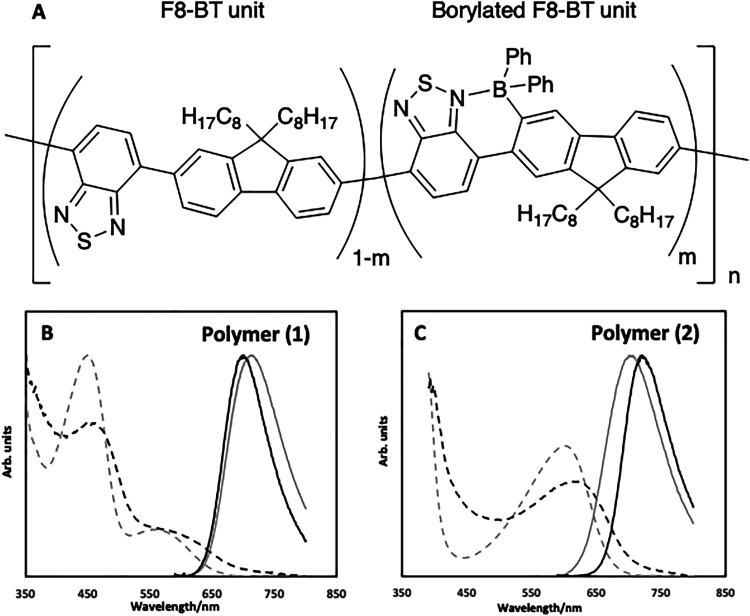
(A) Chemical structures of F8BT and borylated F8BT conjugated polymers (1*m* = 0.25, 2*m* = 1); (B) absorption (dotted lines) and emission (solid lines) of the free polymer (1) in THF (grey) and CPNs in water (black). (C) Absorption (dotted lines) and emission (solid lines) of the free polymer (2) in THF (grey) and CPNs in water (black).

Conjugated polymer nanoparticles were prepared by the modified reprecipitation method reported elsewhere,^22^ utilising borylated poly(9,9-dioctylfluorene-*alt*-benzothiadiazole) as the emitting polymer (Fig. 1A). Two conjugated polymers were used; one composed of 25% borylated units (1, *m* = 0.25), and a second being fully borylated (2, *m* = 1). The effect of adding SPIONs can be seen in the Video S1, where the resulting nanoparticles (using 2) were clearly emissive (as show using an IR responsive camera) whilst responding to an external magnet.

The optical spectra for the particles are shown in Fig. 1B and C. Comparing the CPNs against the free polymers in solution, it can be seen, for (1), there is a slight blue shift in the emission of the polymer when in CPN form with the maximum shifting to 702 nm (Fig. 1B). For the free polymer in a THF solution, the absorption spectra had a clear defined peak at 449 nm, similar to that seen in F8BT,^23^ and a small shoulder at *ca.* 575 nm attributed to the borylated unit, with a similar spectrum observed when in CPN form. For (2), the emission maximum was found to be red shifted to 720 nm for the CPNs, compared to 702 nm for the free polymer in THF (Fig. 1C). The absorption spectra showed an absorption feature at *ca.* 600 nm for both the free polymer and CPN form. Emission quantum yields (QY) of both free polymers in THF were approximately 44%,^14^ which decreased to 2.1% for (1) and 2.7% for (2) when processed into nanoparticles. The decline in emission quantum yield is a direct result of the coiling of the polymer chains, as explored in some depth by Schwartz.^24^

The size, zeta potential and dispersity of the CPNs were determined by dynamic light scattering (DLS), where the particles were found to be between 100 and 120 nm in diameter (Fig. S1) with a polydispersity index between 0.1 and 0.12 and an average zeta potential of −18 mV. A superconducting quantum interference device (SQUID) magnetometer was used to characterise the M–H curves of the CPNs, with the free SPIONs on their own exhibiting saturation of magnetisation (emu g^−1^) of up to 55 emu g^−1^, similar to previous reports.^25,26^ For nanoparticles composed of polymer (1) and SPIONs, we observed magnetisation of up to 10 emu g^−1^, whilst nanoparticles composed of polymer (2) and SPIONs, there was a decrease to 5 emu g^−1^. The reason for the difference between the two magnetization values is currently unclear, although the amount of SPIONs encapsulated by the individual polymers may play an important role.

Transmission electron microscopy (TEM) revealed colloidal structures (Fig. S2), which aggregated when dried on TEM grids displaying varying sizes of the CPNs. As PSMA and the conjugated polymer are composed mostly of carbon, there was little contrast although SPIONs could be observed as black regions encapsulated inside the particles. On average, the particles have a diameter of between 100 and 120 nm, with the SPIONs being *ca.* 5–10 nm in diameter. The slight difference between the sizes of the particles observed on the TEM *versus* the DLS measurements was attributed to the hydrated size of the particles in water.^27^

### Cellular uptake and imaging studies

**Fig. 2:**
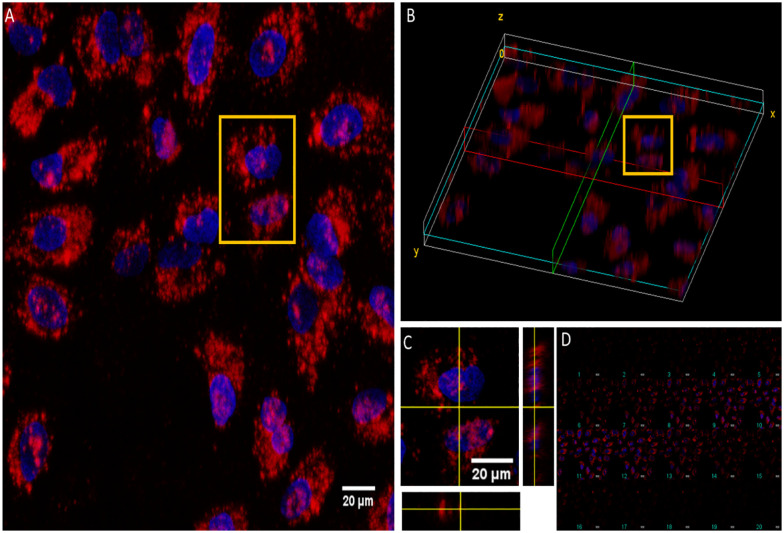
Z-Stack through a HeLa cell after 24 hours of incubation with CPNs prepared from (2) at 1.45 µm steps. Red indicates NPs (excited at 594 nm), whereas blue is DAPI stain (excited at 450 nm). (A) Z-stack at maximum intensity, with yellow box indicating area of interest. (B) Volume view at maximum intensity, with yellow box indicating area of interest. (C) Orthogonal view of Z-stack, with slice 6 taken as showing NP internalised within the cell. (D) Montage of the Z-stack, with 1 indicating the bottom of cell to 20 indicating the top (depth ∼27.8 µm). Scale bar = 20 µm.

To investigate the potential use of the particles in biological imaging, the CPNs described above were initially incubated with HeLa cells at a low concentration (5 µg mL^−1^ of polymer) for 24 hours. The cells were also stained with DAPI and ActinGreen™ ReadyProbes™ Reagent and incubated for 60 mins before imaging (Fig. S3). The CPNs appeared to be taken up by the cells and appeared to be evenly distributed throughout the cytoplasm or located on or near the nucleus (stained blue), they did not seem to affect the cytoskeleton (stained green). To determine whether the nanoparticles were internalised into the cells, CPNs prepared with (2) were incubated with HeLa cells, and 20 optical sections through individual cells were imaged in incremental steps of 27.8 µm from regions around the upper surface of the cell to the lower surface as shown in Fig. 2. One region was highlighted to show that the CPNs were found within the cell and not just on the surface. It was observed from Fig. 2C (6th slice), that the particles were in the same plane as the stained nucleus (blue DAPI stain).

**Fig. 3:**
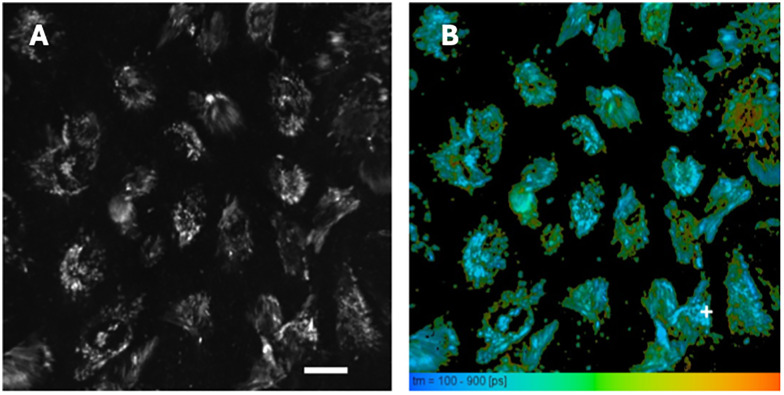
FLIM of HeLa cells using CPNs made from (2). (A) shows a 512 × 512 pixel fluorescence intensity image, using an excitation wavelength of 467 nm and a 500 nm long pass filter. Scale bar 20 µm. (B) shows the corresponding fluorescence lifetime image.

Particles made from polymer (2) had higher quantum yields than particles made from polymer (1), so these were taken forwards to fluorescence lifetime imaging of HeLa cells with as shown in Fig. 3. From the measurements, a triple-exponential fluorescence decay model was found to provide the best fit. The fluorescence lifetime histogram, shown in Fig. S4A, has a peak at around 300 ps, with a shoulder around 500 ps. A representative fluorescence decay is shown in Fig. S4b. It was fitted with a triple exponential fluorescence decay function, with fluorescence decay times of 234 ps (79%), 928 ps (20%) and 5.15 ns (1%), yielding an average fluorescence lifetime of 410 ps. The residuals are flat and the goodness of fit parameter *χ*_R_^2^ = 1.16 as shown in Fig. S4B. Monoexponential or double exponential fluorescence decay functions were not suitable for fitting the fluorescence decay data, as they yielded a much higher *χ*_R_^2^ and residuals that showed systematic deviations around zero, indicating a poor fit.

In conclusion, we report the preparation and use of two aqueous dispersions of deep red-emitting (maxima >700 nm) conjugated polymer nanoparticles (1 and 2) that incorporated superparamagnetic iron oxide particles (SPIONs) and were stabilised by PSMA in cell imaging. The presence of SPIONs allowed for a simple purification of the NPs through magnetism, and the particles exhibited magnetic measurements of up to 10 emu g^−1^. The nanoparticles were capable of being uptaken by HeLa cells, remaining bright within the cells as confirmed by Z-stack imaging and have fluorescence decay profiles which would make them suitable candidates for fluorescence lifetime imaging. The particles have the potential for further use in biological imaging: both as a means for target-specific binding when the surface of the NPs are modified and functionalised; and as a potential multimodal tool utilising the MRI active iron oxide component.

## Conflicts of interest

There are no conflicts of interest.

## Supplementary Material

TB-014-D6TB00192K-s001

TB-014-D6TB00192K-s002

## Data Availability

All data is available from the corresponding author upon reasonable request. Supplementary information (SI) is available. See DOI: https://doi.org/10.1039/d6tb00192k.
