# Supplementary material for: Magnetic borylated conjugated polymer nanoparticles for deep-red optical and lifetime imaging
Source: J Mater Chem B. 2026 Apr 1;14(17):5233–7. doi: 10.1039/d6tb00192k (PMC13058908; doi:10.1039/d6tb00192k)
Supplement: TB-014-D6TB00192K-s001 [file TB-014-D6TB00192K-s001.pdf]

Supplementary Information (SI) for Journal of Materials Chemistry B.
This journal is © The Royal Society of Chemistry 2026

Title: Magnetic borylated conjugated polymer nanoparticles for deep-red optical and lifetime imaging.

Authors: Struan Bourke[1], Yurema Teijeiro Gonzalez[1], Patrick Bergstrom Mann[1], Daniel Luke Crossley[2], Laura Urbano[3,4], Lea Ann Dailey [5], Klaus Suhling[1], Michael L. Turner[2], Michael J. Ingleson[2,6] and Mark Green*[1].

1) Department of Physics, King's College London, Strand Campus, London WC2R 2LS, United Kingdom.
2) School of Chemistry, University of Manchester, Manchester M13 9PL, United Kingdom.
3) Institute of Pharmaceutical Sciences, King's College London, Waterloo Campus, London SE1 9NH, United Kingdom.
4) School of Health, Medicine and Life Sciences, School of Life and Medical Sciences, University of Hertfordshire, Hatfield, AL10 9AB, United Kingdom.
5) Department of Pharmacy, University of Vienna, Josef-Holaubek Platz2 (UZA II), 1090

   Wien.
6) Department of Chemistry, University of Edinburgh, Joseph Black Building, David Brewster Road, Edinburgh, EH9 3FJ, United Kingdom.

Experimental Method

Materials

The two borylated poly(9,9-dioctylfluorene-alt-benzothiadiazole) (F8-BT), **(1)** and **(2),** were prepared as described previously.[1] Poly(styrene-co-maleic anhydride), cumene terminated (PSMA) (MW= 1,700), tetrahydrofuran (THF), and magnetic iron oxide nanoparticles (10-40 nm, 0.8-1.4% total solid in heptane) were purchased from Sigma-Aldrich (England, UK). Blue/Green, ActinGreen ™ ReadyProbes™ Reagent and 4′,6-Diamidine-2′-phenylindole (DAPI) were purchased from Thermo Fisher Scientific (England, UK) and used as received.

Preparation of conjugated polymer nanoparticles

Deep-red emitting CP nanoparticles.

The method was adapted from work by Wu *et al.*[2] In a typical synthesis, 10 mg of **(1)** or **(2)** and 10 mg of PSMA were added to separate vials and dissolved in 10 mL THF (1 mg/mL) followed by sonication for 10 minutes. 0.5 mL of either **(1)** or **(2)** stock and 0.25 mL of PSMA stock were added to 0.20 mL THF. In a separate container, 5 mL of the iron oxide nanoparticles solution (11.7 – 20 mg/ml) was taken, and the heptane removed before being re-suspending in 5 mL THF. Of this, 0.05 mL of these was added to the solution containing the conjugated polymer and PSMA to give a total volume of 1 mL. The solution was then injected into 9 mL deionised water and sonicated for 10 minutes. The solution was then stirred continuously at 400 rpm, at room temperature, for 24 hours to evaporate off THF. The loss of water was compensated by readjustment to 10 mL. The nano-suspension (50 µg/mL of either **(1)** or **(2)** or total solid 0.134 - 0.175 mg/mL) was placed against a 1 kg neodymium magnet for 24 hours. Non-magnetised NPs were removed, and the particles were re-suspended in 1 ml deionised H_2O and stored at room temperature.

CPNs Optical and Physical Measurements

Absorption spectra were measured using a Hitachi U-4100 UV-Visible-NIR spectrometer using a 1 cm path length quartz cuvette. Photoluminescence spectra were measured using a Horiba Fluoromax-4 spectrofluorometer. Particle size distributions and zeta potentials were obtained using a Malvern Zetasizer utilising dynamic light scattering. Transmission electron microscopy images were acquired on a JEOL JEM 1400Plus with filament electron source at 120 kV at the Centre of Ultrastructural Imaging, King's College London. Image analysis was performed with ImageJ software. The QY were measured using the dye comparison method.[3] Photoluminescent quantum yields (PLQYs) were estimated using indocyanine green (ICG, PLQY= 12%) in DMSO as a standard[4], according to the following equation:

$$\Phi_{sample} = \Phi_{ref}\frac{I_{sample}\ OD_{ref}\ \eta_{sample}^2}{I_{ref}\ OD_{sample}\ \eta_{ref}^2}$$

Where the subscripts *sample* and *ref* note sample and reference fluorophore respectively, *Φ* is the fluorescence quantum yield, *I* is the integrated sample fluorescence, *OD* is the optical density at the chosen excitation wavelength and *η* is the refractive index of the solvent. To avoid self-absorption effects, the absorbances of the sample and reference fluorophore solutions at the excitation wavelength were below 0.1.

Magnetic measurements were done at the London Centre of Nanotechnology using a physical property measurement system (PPMS, Quantum design). The samples, 3.4 mg of the SPIONs, 0.1 mg of 2:1 CP:PSMA, 0.1 mg of 1:5 CP:PSMA and 0.5 mg of 1:10 CP:PSMA, were weighed out and analysed by measuring the M-H loops in an applied field ranging from -2k Oe to 62k Oe at 310K (36.5°C).

Cell Culture

HeLa cells were verified as HeLa by STR profiling from Eurofins MWG. HeLa cells were grown at 37 °C in complete DMEM (Invitrogen) supplemented with 10% foetal bovine serum (FBS - Sigma) and 1 % Penicillin-Streptomycin (PenStrep - Invitrogen) in T75 tissue culture flask (Helena TTP).

Nanoparticle treatment

HeLa cells were grown on 1.5 µ-plate 8 well plate (ibidi) overnight at 30,000 cells/ml. Cells were treated with NPs, negative and positive controls for 24h. The **(1)** and **(2)** nanoparticle suspension was serially diluted in DMEM to have a polymer concentration of 5 µm/mL. 150 µl of the CN-FO-DPD nanoparticle suspension was added to 150 µL of the aforementioned media (for the 8 well plate). These were incubated for 24 hours prior to fixation for immunofluorescence or live cell imaging.

Immunofluorescence

After incubation, cells were fixed with 4% paraformaldehyde in phosphate buffered saline (PBS, Sigma Aldrich) for 10 min at room temperature; cells were permeabilised by incubation with 0.3% Triton X-100 in blocking buffer (0.5% bovine serum albumin, 0.1% NaN_3 in PBS) for 5 min followed by three 5 minutes incubations in blocking buffer plus 20 mM glycine. Plates were incubated with DAPI at 1 µl/mL. and ActinGreen ™ ReadyProbes™ Reagent for 1 h at room temperature. The plates were washed three further times with blocking buffer and left in PBS.

Fluorescence Microscopy and Image Processing

FLIM measurements were taken with a TCSPC (Time-Correlated Single Photon Counting) card in combination with an inverted confocal microscope (Leica TCS SP2). The system was excited with a picosecond diode laser (Hamamatsu PLP-10 470) at 467 nm and a repetition rate of 10 MHz. The fluorescence emission was detected by a GaAsP hybrid detector (Becker & Hickl HPM-100-40 based on an R10467-40 GaAsP hybrid photomultiplier) prior collected by the TCSPC module (SPC-150) and the excitation light was discriminated from the emission by using a 500LP filter. A RSP 500 excitation beam splitter and a 63× 1.2 N.A. water-immersion objective was used to acquire the images. The line scan speed was set to 400 Hz, the image size to 512 × 512 pixels with a pixel size of 470 × 470 nm^2 (yielding a 241 µm × 241 µm field of view) and a pinhole of 2 Airy units. The collected data was analysed with SPCImage.

The fluorescence lifetime τ_f is the average time a fluorophore remains in the excited state. It is given by

$$\tau_f = \frac{1}{k_r + k_{nr}}$$

Where k_r is the radiative rate constant and k_{nr} is the non-radiative rate constant. k_r depends on the refractive index of the surrounding environment, and k_{nr} on non-radiative decay processes. In addition, this parameter does neither depend on the intensity nor the fluorophore concentration. This is why Fluorescence Lifetime IMaging (FLIM) has been carried out in order to investigate the nanoparticles fluorescence lifetime in HeLa cells.

Several single intensity decays were taken for the nanoparticles of (1) and (2), where FLIM images were acquired. The fluorescence lifetime values were determined by fitting the intensity decays with a multi-exponential model such as:

$$I(t) = \sum_i \alpha_i exp(- t/\tau_i)$$

Where α_i corresponds to each one of the amplitude contributions and τ_i to the i_{th} fluorescence lifetime. $\sum \alpha_i$ is normalised to 1.

Live cells were imaged on an inverted confocal microscope (Leica TCS SP2) and an internal analogue photomultiplier tube detector, whose detection wavelength range for the fluorescence

emission was set to 600-750 nm. A RSP 500 excitation beam splitter and a 63× 1.2 N.A. water-immersion objective was used to acquire the images. The line scan speed was set to 400 Hz, the image size to 512 × 512 pixels with a pixel size of 470 × 470 nm^2 and a pinhole of 2 Airy units. Original data were processed using ImageJ. Figures and video were both generated using ClearVolume and ImageJ 3D viewer.

Deep-red Imaging

Raspberry Pi NIR imaging was carried out using a Raspberry Pi "NoIR" camera module (#2357308, Farnell UK) and Raspberry Pi 2 (model B, #2461030, Farnell UK), in conjunction with a 715 nm long pass coloured glass filter (FGL715, Thorlabs UK). Sample excitation was achieved with ambient light (between 400 to 700 nm) in the laboratory.

Statistical Analysis

For spectra, quantum yields and size values from DLS all experiments were performed in triplicate with the data presented as average. Bar graphs were drawn using Origin Statistical significance was assessed using an unpaired two-tailed Student's t-test assuming unequal variance.

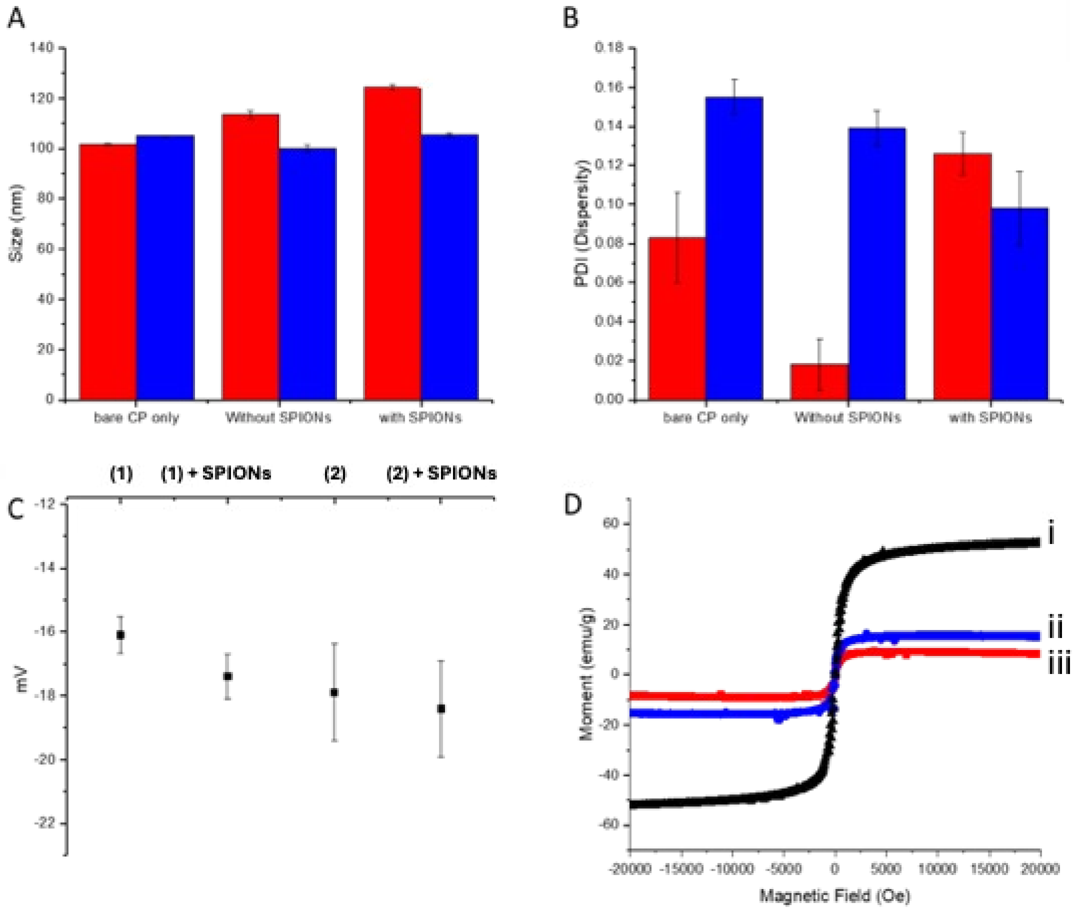

Supporting figure 1. Different graphs of physical characteristics by dynamic light scattering. **(1)** NPs are shown in red, and **(2)** NPs are shown in blue. A) Graph of CPs vs. mean diameter/size (nm) of the CPNs. B) Graph of the different CP ratio vs PDI. C) Graph of different CPNs vs zeta potential (mV) including those not containing SPIONs. D) Magnetic measurements from the SQUID magnetometer, showing the M-H loops at 310 K of different CPNs. i) SPIONs used in the experiments containing no PSMA or CP; (ii) NPs consisting of CP **(2)**, PSMA and SPIONs; (iii) NPs consisting of CP **(1)**, PSMA and SPIONs.

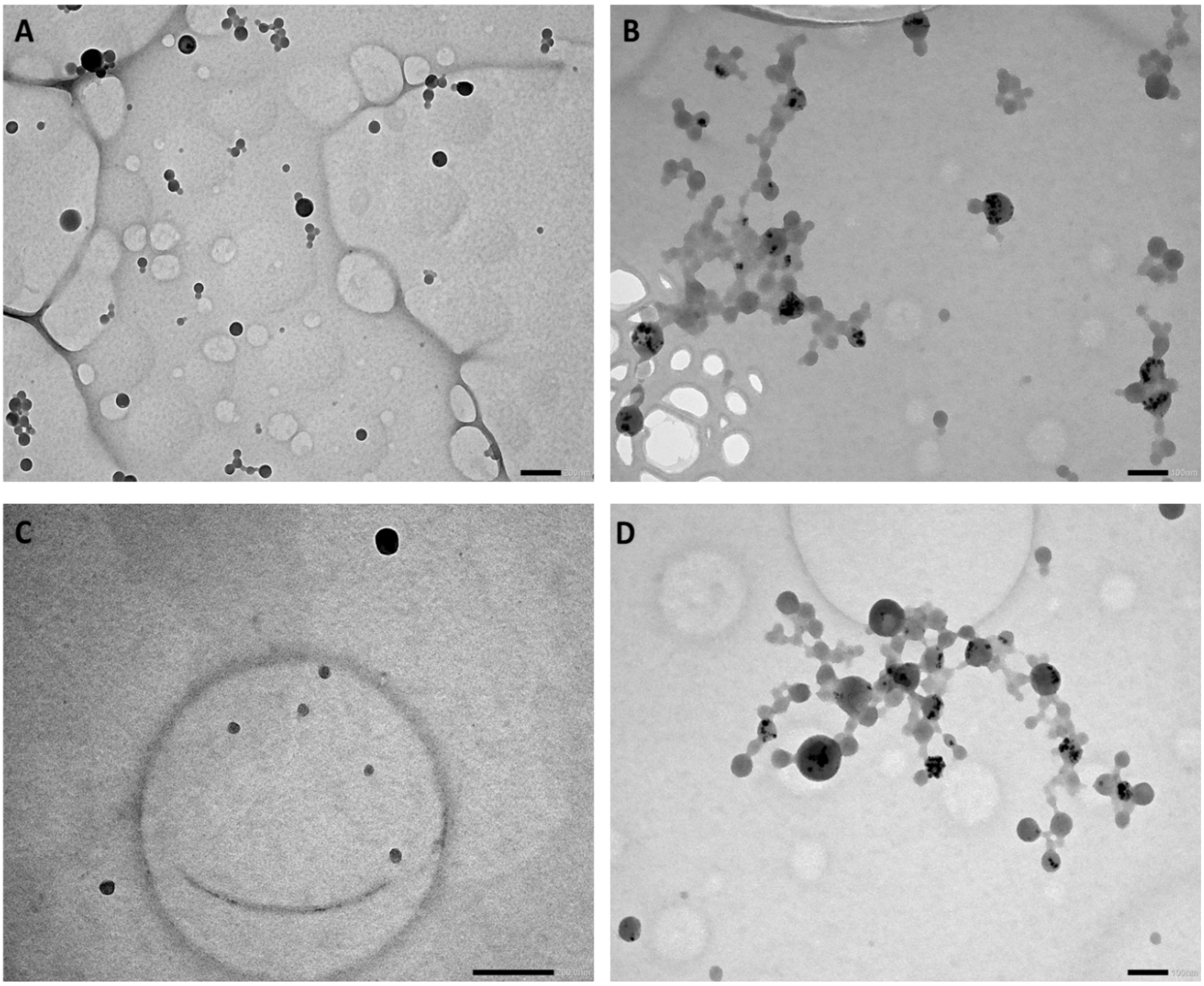

Supporting figure 2. TEM images of the **(1)** NPs, A) without SPIONs and B) with SPIONs. **(2)** NPs are shown, C) without SPIONs and D) with SPIONs, A) and C) were taken at x20K (scale bars = 200 nm) whereas B) and D) were taken at 40K (scale bars = 100 nm)

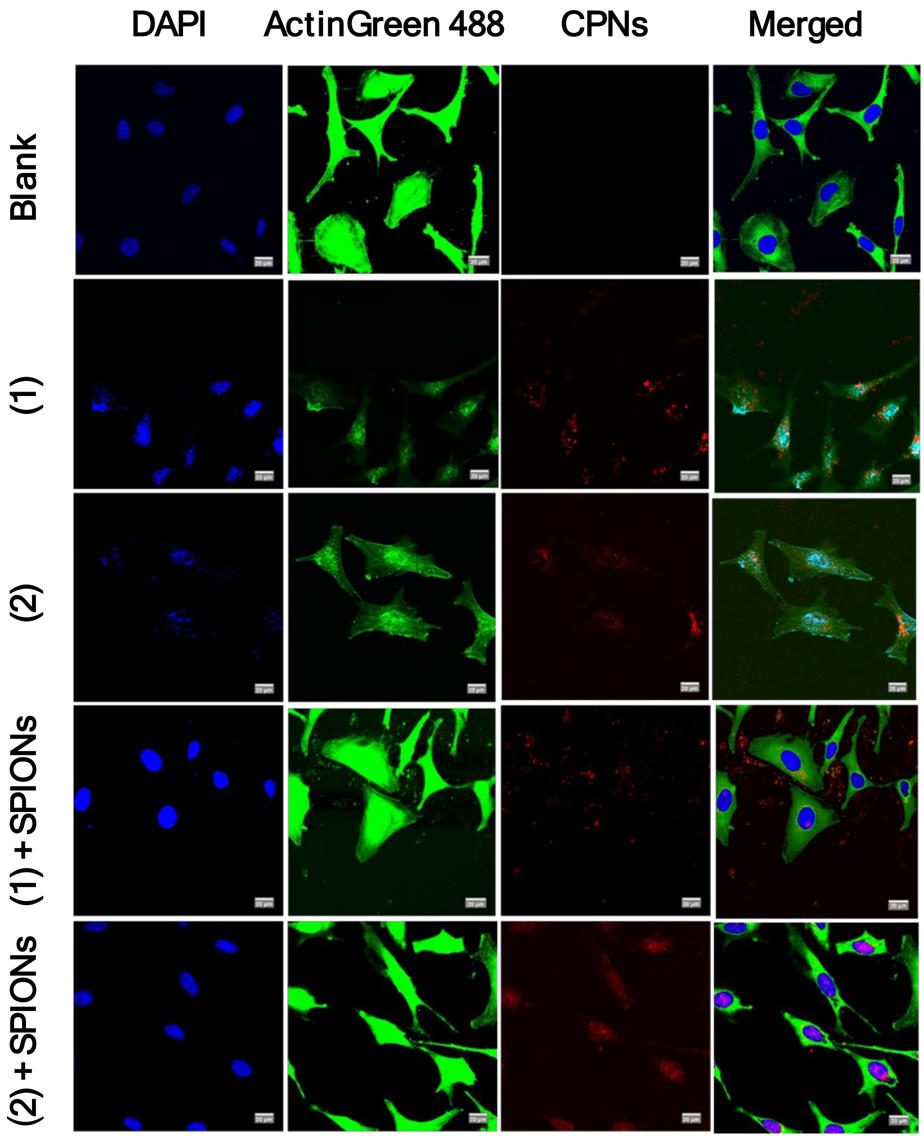

Supporting figure 3. Representative epifluorescence images of HeLa cells fixed and stained with DAPI (blue) to visualise DNA, respectively, 24 h after nanoparticle treatment (Red). The first row shows PMSA NPs, which do not fluoresce, whereas both the CPs with and without SPIONs fluoresce red. Scale bar 20 μm.

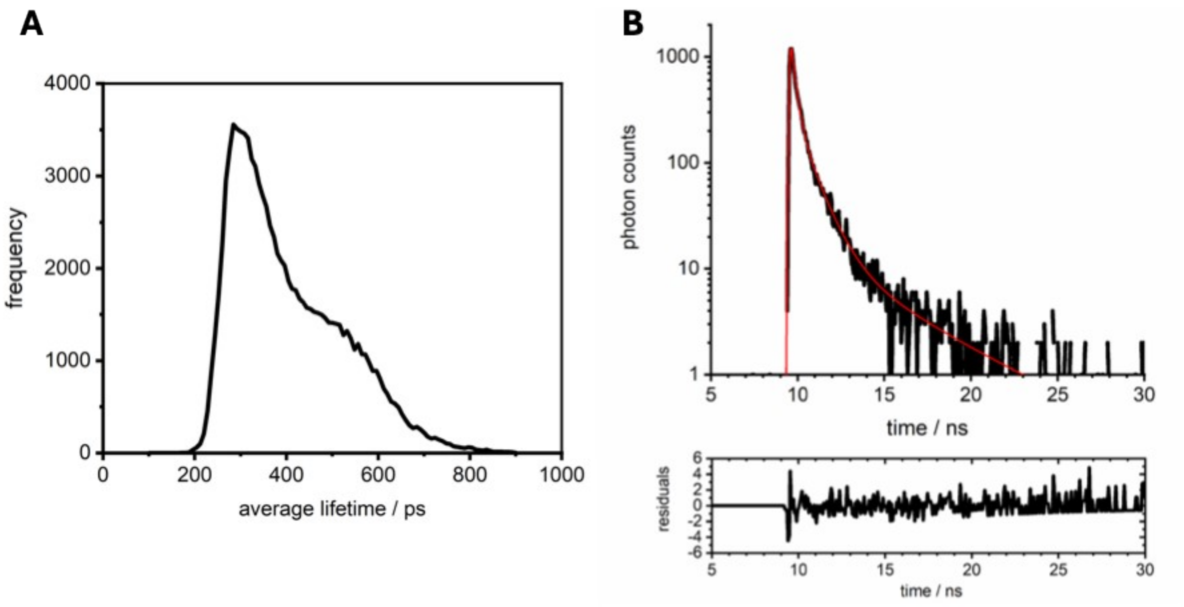

Supporting figure 4. FLIM of HeLa cells using CPNs made from (**2**). A) shows the average fluorescence lifetime histogram. B) shows a representative fluorescence decay, taken from a 5 × 5 pixel area indicated by the white cross in the bottom right-hand corner of 3B). The fluorescence decay was fitted with a triple-exponential decay function, yielding an average fluorescence lifetime of 410 ps.

Supporting video 1. Movie of the response of magnetic CPNs in solution made from **(2)** when exposed to a Nd magnet, using a IR sensitive camera. The particles had previously been collected *via* magnet to the side of the vial (right hand side) before the video was filmed.

References

1) Crossley, D. L.; Urbano, L.; Neumann, R.; Bourke, S.; Jones, J.; Dailey, L. A.; Green, M.; Humphries, M. J.; King, S. M.; Turner, M. L.; Ingleson, M. J. Post-polymerization C-H borylation of donor-acceptor materials gives highly efficient solid state near-infrared emitters for near-IR-OLEDs and effective biological imaging. *ACS Appl. Mater. Interfaces* **2017**, *9* (34), 28243–28249.

2) Wu, C.; Hansen, S. J.; Hou, Q.; Yu, J.; Zeigler, M.; Jin, Y.; Burnham, D. R.; McNeill, J. D.; Olson, J. M.; Chiu, D. T. Design of highly emissive polymer dot bioconjugates for in vivo tumor targeting. *Angew. Chemie - Int. Ed.* **2011**, *50*, 3430–3434.

3) Levitus, Marcia. 'Tutorial: Measurement of Fluorescence Spectra and Determination of Relative Fluorescence Quantum Yields of Transparent Samples'. *Methods and Applications in Fluorescence* 8, no. 3 (April 2020): 033001.

4) Benson, R. C., and H. A. Kues. 'Fluorescence Properties of Indocyanine Green as Related to Angiography'. *Physics in Medicine & Biology* 23, no. 1 (January 1978): 159.
